# Impacts of Mn, Fe, and Oxidative Stressors on MnSOD Activation by AtMTM1 and AtMTM2 in *Arabidopsis*

**DOI:** 10.3390/plants11050619

**Published:** 2022-02-24

**Authors:** Shu-Hsuan Hu, Tsung-Luo Jinn

**Affiliations:** Institute of Plant Biology and Department of Life Science, National Taiwan University, Taipei 10617, Taiwan; d98b42003@ntu.edu.tw

**Keywords:** Fe/Mn ratio, MnSOD, mitochondrial carrier protein, Mn transporter, SOD, superoxide

## Abstract

It has been reported that the mitochondrial carrier family proteins of AtMTM1 and AtMTM2 are necessary for manganese superoxide dismutase (MnSOD) activation in *Arabidopsis*, and are responsive to methyl viologen (MV)-induced oxidative stress. In this study, we showed that MnSOD activity was enhanced specifically by Mn treatments. By using *AtMnSOD*-overexpressing and *AtMnSOD*-knockdown mutant plants treated with the widely used oxidative stressors including MV, NaCl, H_2_O_2_, and tert-butyl hydroperoxide (t-BH), we revealed that *Arabidopsis* MnSOD was crucial for root-growth control and superoxide scavenging ability. In addition, it has been reported that *E*. *coli* MnSOD activity is inhibited by Fe and that *MTM1*-mutated yeast cells exhibit elevated Fe content and decreased MnSOD activity, which can be restored by the Fe^2+^-specific chelator, bathophenanthroline disulfonate (BPS). However, we showed that BPS inhibited MnSOD activity in *AtMTM1* and *AtMTM2* single- and double-mutant protoplasts, implying that altered Fe homeostasis affected MnSOD activation through AtMTM1 and AtMTM2. Notably, we used inductively coupled plasma-optical emission spectrometry (ICP-OES) analysis to reveal an abnormal Fe/Mn ratio in the roots and shoots of *AtMTM1* and *AtMTM2* mutants under MV stress, indicating the importance of AtMTM1 in roots and AtMTM2 in shoots for maintaining Fe/Mn balance.

## 1. Introduction

Superoxide dismutases (SODs) are distributed in the cytoplasm, chloroplasts, and mitochondria of prokaryotic and eukaryotic cells [1,2,3]. They are classified as CuZnSOD, FeSOD, MnSOD, or NiSOD according to the transition metal cofactor ions at the active site [4,5,6]. Cellular superoxide (O_2_^•−^) is mainly generated from electron transport chain complexes, and SODs catalyse the dismutation of O_2_^•−^ to O_2_ and H_2_O_2_. Toxic H_2_O_2_ is converted to H_2_O mainly by catalase, ascorbate peroxidase, and glutathione peroxidase [7]; thus, SODs cooperate with other enzymes to relieve oxidative stress [8,9,10,11]. It has been reported that *AtMnSOD*-overexpressing plants exhibit increased catalase and peroxidase activities, with decreased malondialdehyde content after NaCl treatment, and maintain a higher germination rate in the presence of oxidative stressors, such as methyl viologen (MV) and H_2_O_2_ [12,13]. Plants harboured with antisense *AtMnSOD* show decreased MnSOD protein levels, with altered tricarboxylic acid cycle enzyme levels and root growth after treatment with NaCl, sorbitol, Fe, and MV [14]. In this study, we established both *AtMnSOD*-overexpressing (*MnSOD-OE*) and *AtMnSOD*-knockdown (*msd1*) plants to confirm the cofactor specificity and post-translational regulation of MnSOD under conditions of oxidative stress. In addition, antagonism between Fe and Mn has been reported in tomato and rice plants, in which Fe suppresses Mn levels and vice versa [15,16,17]. Therefore, we also investigated the effect of metal ion treatments on MnSOD activity in *Arabidopsis*.

The MnSOD dimer in *E. coli* is localised in the cytosol and is regulated by repressors and Fe ion concentrations. Fe suppresses the biosynthesis of MnSOD at the transcriptional and post-translational levels [18,19]. *E. coli* MnSOD protein and activity levels are inhibited by Fe^2+^, but not Co^2+^, Ni^2+^, or Zn^2+^, and both protein and activity levels are induced by Mn^2+^ treatment. It has been suggested that Fe and Mn compete for the metal-binding site of MnSOD, but only Mn can activate the enzyme [19]. X-ray crystallography further shows that MnSOD active sites bind Mn or Fe, but Fe-substituted MnSOD blocks the substrate access channel and inactivates the enzyme [20,21]. Moreover, intracellular O_2_^•−^ inactivates Fe-S cluster biogenesis enzymes [22], and the interruption in the mitochondrial Fe-S pathway is associated with MnSOD activity in *E. coli* [23,24].

Yeast Mn transporters of plasma membrane-localized SMF1, intracellular vesicle-localized SMF2, and mitochondrial inner membrane-localized carrier protein MTM1 are involved in mitochondrial MnSOD activation [25,26,27,28]. The yeast *MTM1* mutant retains normal MnSOD protein levels after treatment with the metals Mn, Fe, Cu, and Zn, but MnSOD activity is only restored by Mn treatment [25]. Yeast MnSOD acquires its catalytic cofactor, Mn, via MTM1; thus, MnSOD monomers fold into active tetrameric enzymes during post-translational regulation, and unknown factors may also facilitate Mn binding [27]. Intriguingly, yeast *MTM1* mutant retains normal mitochondrial Mn levels and exhibits higher mitochondrial Fe content [25]. In addition, *MTM1*- and *SMF2*-mutated yeast cells exhibit normal MnSOD protein levels, and the loss of MnSOD activity is fully restored by Mn supplementation [25,27]. Although the yeast *SMF2* mutant exhibits decreased Mn content and lower MnSOD activity, mitochondrial Fe levels in the mutant are normal. However, treatment with the Fe^2+^-specific chelator, bathophenanthroline disulphonate (BPS), decrease mitochondrial Fe levels and increases MnSOD activity [29]. BPS treatment also restores MnSOD activity in the yeast *MTM1* mutant [29]. Taken together, these data indicate that the relationship between altered mitochondrial Fe levels and MnSOD activity in yeast is unclear.

*Arabidopsis* mitochondrial carrier proteins, AtMTM1 and AtMTM2, bind Mn for mitochondrial MnSOD activation. These proteins share a high amino acid sequence homology with yeast MTM1 and they are induced by MV [30,31]. Our previous study showed that AtMTM2 has distinct expression levels from AtMTM1 during development, and that Mn levels are lower in the roots of both *mtm1* (miRNA-mediated *AtMTM1*-knockdown) and *mtm2* (T-DNA insertional *AtMTM2*-knockout) mutants. However, Fe levels are decreased in the roots of the *mtm1* mutant, but remain normal in the *mtm2* mutant [30]. The role of Mn transporters in plant mitochondria is unclear [32,33], and the effects of Fe metabolism and mitochondrial Fe-S cluster biogenesis proteins on MnSOD activation in *Arabidopsis* have not been elucidated.

In this study, we observed that MnSOD activity was enhanced significantly with higher Mn concentrations, but not with Fe, Cu, or Zn treatments, implying Mn cofactor specificity for MnSOD activation. Moreover, *AtMTM1* and *AtMTM2* gene expression levels increased with *MnSOD* gene expression levels in the presence of the commonly used oxidative stressors including MV, NaCl, H_2_O_2_, and tertiary-butyl hydroperoxide (t-BH). We showed MnSOD activity in *MnSOD-OE* and *msd1* mutants corresponded to the representative treatments of MV and NaCl, and revealed MnSOD was crucial for early root growth and plant development. In addition, we treated *mtm1*, *mtm2,* and *mtm1 mtm2*-double mutant with Mn supplementation and the Fe chelator, BPS, and found that Mn and Fe homeostasis affected the primary root length and MnSOD activity via AtMTM1 and AtMTM2. Moreover, Fe/Mn ratio analysis further demonstrated the physiological importance of AtMTM1 in roots and AtMTM2 in shoots for maintaining the Fe/Mn balance under the representative MV treatment.

## 2. Results

### 2.1. Generation of AtMnSOD-Overexpressing Plants and Characterisation of AtMnSOD T-DNA Insertion Mutants

To increase the effect of Mn, Fe, and oxidative stressors on *Arabidopsis AtMnSOD*, *AtMTM1*, and *AtMTM2* expression levels, we generated *AtMnSOD*-overexpressing plants (*MnSOD-OE*; MnSOD-apoprotein-overexpressing plants) and characterised the *AtMnSOD* T-DNA insertion (*msd1*) mutants (Supplementary Figures S1 and S2).

The highest expression line of *MnSOD-OE* plants with approximately two-fold *MnSOD* mRNA expression levels was used in the following study; however, MnSOD activity and protein levels in *MnSOD-OE* plants were not significantly affected. *MnSOD-OE* plants showed a late-flowering phenotype compared to wild-type (Col) plants (Supplementary Figure S1).

*msd1* plants were characterized by genotyping and RT-qPCR. We confirmed that *msd1* is a heterologous T-DNA insertional knockdown mutant. The lowest expression line with a 20% reduction in *MnSOD* transcript level was used in the following study. MnSOD activity and protein levels in the *msd1* mutant were lower, while the *msd1* mutant showed an early flowering phenotype compared to Col plants (Supplementary Figure S2). Moreover, we are unable to screen the homologous *msd1* plants, implying the lethal effect of the null mutant for plant germination.

### 2.2. Effect of Mn and Fe on Transgenic AtMnSOD-Overexpressing Plants

**Figure 1:**
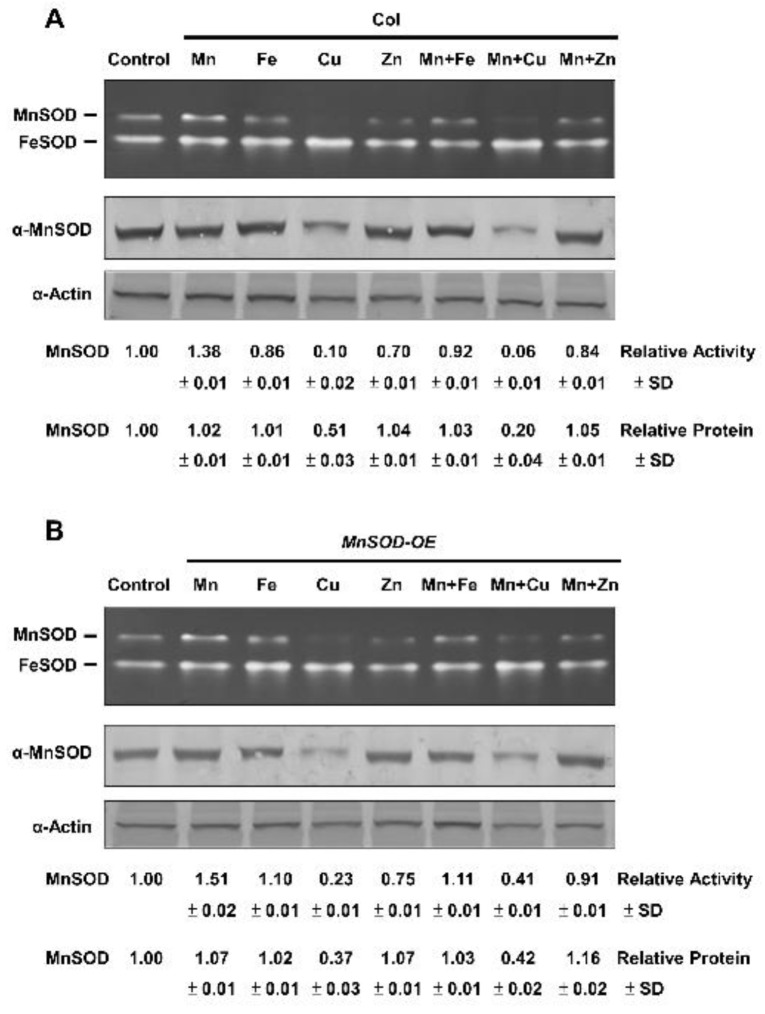
MnSOD activity and protein levels of Col and *MnSOD-OE* plants in response to Mn, Fe, and duplex metal ion treatments. (**A**,**B**) Fourteen-day-old seedlings were treated with 1 mM of Mn, Fe, Cu, Zn, Mn and Fe, Mn and Cu, or Mn and Zn for 16 h. In-gel SOD activity assay (**top**) and immunoblotting with α-MnSOD and α-Actin antibodies (**bottom**) were conducted. Actin was used as a loading control. MnSOD activity and protein levels were measured relative to those in control. Data represent one of three independent repeats.

MnSOD activity in Col and *MnSOD-OE* plants was analysed. Fourteen-day-old seedlings were treated with 1 mM of the metal ions, Mn, Fe, CuSO_4_ (Cu), ZnSO_4_ (Zn), Mn and Fe, Mn and Cu, or Mn and Zn, for 16 h and analyzed by in-gel SOD activity assay (Figure 1). We observed that MnSOD activity was increased after Mn treatment, but was decreased after Fe, Cu, and Zn treatments in Col plants. Treatments of Mn and Fe, Mn and Cu, or Mn and Zn caused intermediate MnSOD activity in Col plants (Figure 1A), indicating that antagonisms between Mn and other metals occurred. These effects were obvious in *MnSOD-OE* plants (Figure 1B), implying an increase in the activation of MnSOD-apoprotein after Mn treatment in *MnSOD-OE* plants. Moreover, the protein level in *MnSOD-OE* and Col plants is similar, implying the saturated level of MnSOD protein inside cells.

### 2.3. Post-Translational Regulation of MnSOD through Oxidative Stressors

*AtMTM1* is elevated in response to MV, but not H_2_O_2_, when seedlings are grown on plates containing these stressors [31], and we have reported that *AtMTM1*, *AtMTM2*, and *MnSOD* gene expression levels increase after MV treatment [30]. In this study, 14-day-old Col seedlings in 1/2 MS liquid medium were treated with the widely used oxidative stressors including 5 μM MV, 150 mM NaCl, 10 mM H_2_O_2_, or 1 mM tert-butyl hydroperoxide (t-BH) with agitation for 2 to 8 h.

**Figure 2:**
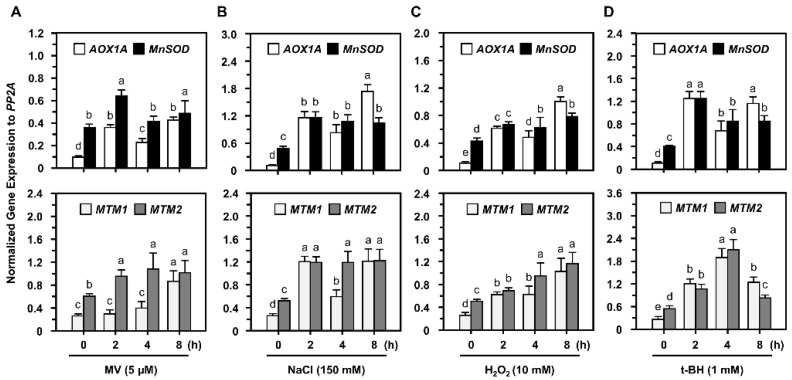
*AtMnSOD*, *AtMTM1*, and *AtMTM2* gene expression levels of Col in response to oxidative stressors. (**A**–**D**) Fourteen-day-old seedlings were incubated with 5 μM MV, 150 mM NaCl, 10 mM H_2_O_2_, or 1 mM t-BH for 2 to 8 h. Gene expressions were normalized to *AOX1A* which is an oxidation-responsive gene. *PP2A* was an input control. Data are mean ± SD of three biological replicates. The statistical significances (*p* < 0.05) are indicated as different letters (Duncan’s multiple range test).

RT-qPCR analysis showed that *AtMTM1*, *AtMTM2*, and *MnSOD* gene expression levels were elevated as early as 2 h after all oxidative stress treatments (Figure 2), and we applied MV or NaCl as the representative stressors in the following studies. The mitochondrial oxidation-responsive gene, *AOX1A*, was used as a reference, as previously reported [30]. *AtMTM1* and *AtMTM2* expression levels in control without stressors are shown in Supplementary Figure S3. Our results implied that oxidative stressors specifically induced *AtMTM1* and *AtMTM2*.

**Figure 3:**
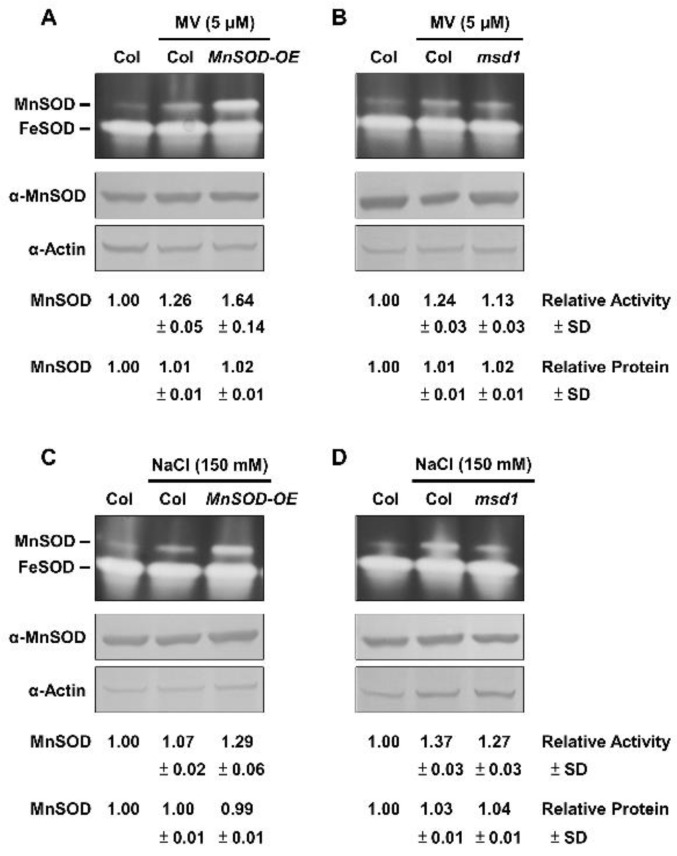
MnSOD activity and protein levels of *MnSOD-OE* and *msd1* seedlings in response to oxidative stressors. Fourteen-day-old seedlings were incubated with 5 μM MV (**A**,**B**) or 150 mM NaCl (**C**,**D**) for 24 h, as indicated. In-gel SOD activity assay (**top**) and immunoblotting with α-MnSOD and α-Actin antibodies (**bottom**) were conducted. Actin was used as a loading control. MnSOD activity and protein levels were measured relative to those in Col control. Data represent one of three independent repeats.

We showed that MnSOD activity was similar between Col and *MnSOD-OE* plants, and was lower in *msd1* plants without oxidative stressors (Supplementary Figures S1 and S2). To clarify the physiological role of MnSOD under oxidative stress, we applied two representative oxidative stressors of MV and NaCl. We incubated 14-day-old seedlings of Col, *MnSOD-OE*, and *msd1* in 1/2 MS liquid medium containing 5 μM MV or 150 mM NaCl with agitation for 24 h, and measured MnSOD activity and protein levels (Figure 3). MnSOD activity was markedly induced in *MnSOD-OE* and was slightly induced in *msd1* plants by MV stressor, and MnSOD activity was still lower in *msd1* plants than in Col plants under stress (Figure 3A,B); however, MnSOD protein levels were not significantly different. Similar expression patterns were observed in Col, *MnSOD-OE*, and *msd1* seedlings under NaCl stress (Figure 3C,D). These results indicated the post-translational regulation of MnSOD under oxidative stress in *Arabidopsis*.

### 2.4. Role of MnSOD in the Control of Primary Root Growth during Oxidative Stress

**Figure 4:**
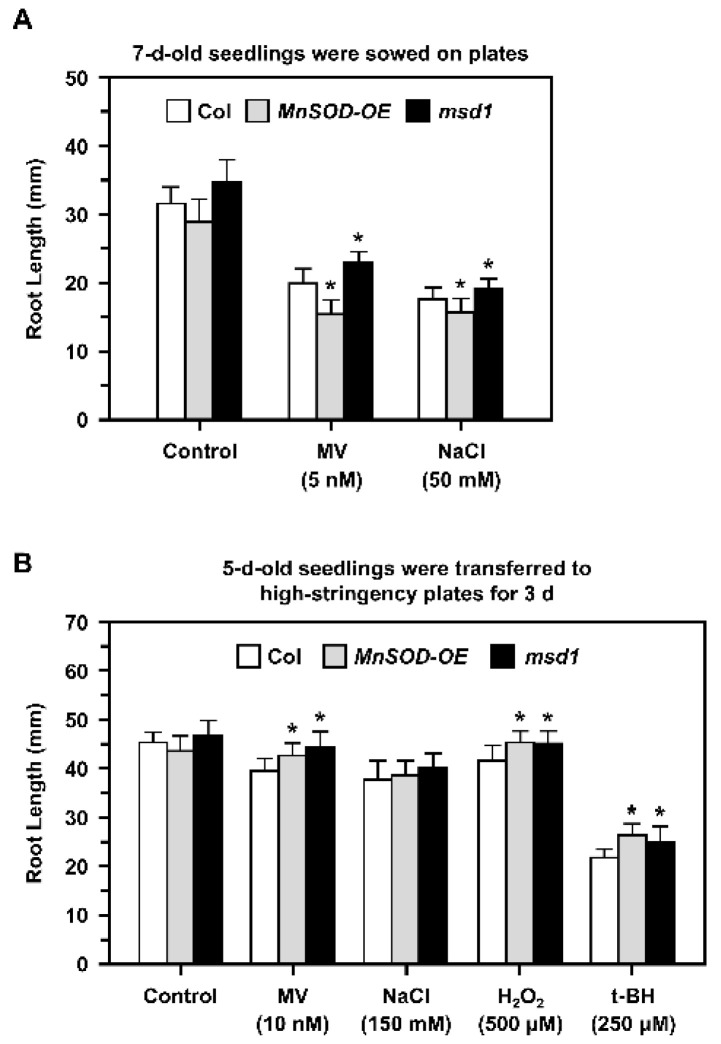
Root lengths of Col, *MnSOD-OE*, and *msd1* seedlings during oxidative stress. (**A**) Seedlings were sown on 1/2 MS plates containing 5 nM MV and 50 mM NaCl for 7 days, and the root lengths were measured. (**B**) Five-day-old seedlings with similar root lengths were transferred from 1/2 MS medium to high-stringency plates containing 10 nM MV, 150 mM NaCl, 500 μM H_2_O_2_, or 250 μM t-BH for 3 days, and the root lengths were measured. Data are mean ± SD of three independent repeats. *n* = 30 seedlings. * Significant at *p* < 0.05 compared with the Col.

A previous study of the *Arabidopsis MnSOD*-knockdown mutant *oiwa* showed that it is a female gametophytic mutant with defective embryo sac development and fertilization, and that the mutation affects reactive oxygen species homeostasis in the mitochondria and cytosol [34,35]. In this study, we focused on the role of MnSOD in early root growth under stress conditions. We applied different stressors in 1/2 MS plates and measured the primary root lengths of Col, *MnSOD-OE*, and *msd1* seedlings (Figure 4)**.**

When seedlings were grown on plates containing 5 nM MV or 50 mM NaCl (Figure 4A), root growth was inhibited in all seedlings. In addition, 7-day-old *MnSOD-OE* seedlings had shorter roots and *msd1* had longer roots compared to the roots of Col plants.

Five-day-old seedlings with similar root lengths were then transferred from 1/2 MS plates to high-stringency plates containing 10 nM MV, 150 mM NaCl, 500 μM H_2_O_2_, or 250 μM t-BH for 3 days (Figure 4B). Root growth was slightly inhibited by MV, NaCl, and H_2_O_2_, and was markedly inhibited by t-BH. In addition, both *MnSOD-OE* and *msd1* seedlings had longer roots in the presence of MV, H_2_O_2_, and t-BH, and this may be restricted to the shorter period of treatment. Taken together, we noticed that MnSOD responded to stress conditions during early primary root growth.

### 2.5. O_2_^•−^ and H_2_O_2_ Accumulation and Distribution in AtMnSOD-Overexpressing Plants under Stress

**Figure 5:**
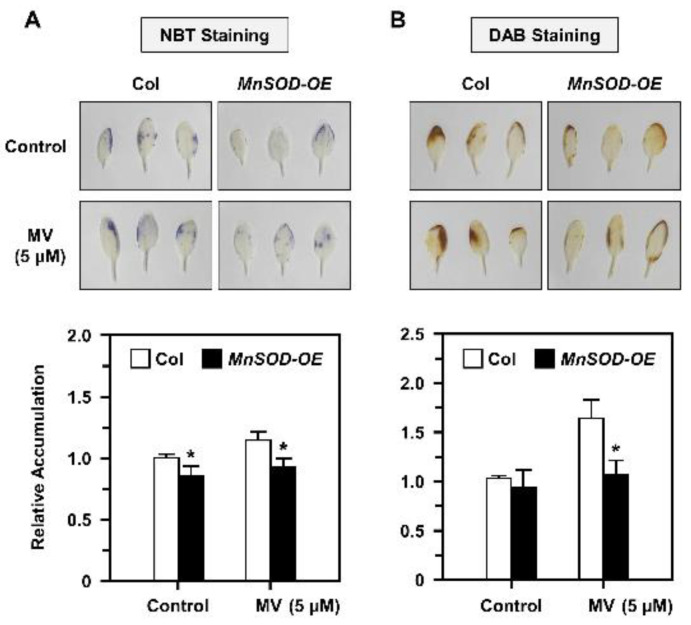
O_2_^•−^ and H_2_O_2_ accumulation of Col and *MnSOD-OE* seedlings under MV stress. (**A**,**B**) Twenty-one-day-old seedlings were treated with 5 μM MV for 3 days, then O_2_^•−^ and H_2_O_2_ accumulation were analyzed by NBT and DAB staining (**top**), respectively. Each accumulation in a plant was measured relative to Col (**bottom**). Data are mean ± SD of three independent repeats. * Significant at *p* < 0.05 compared with the Col.

The O_2_^•−^ and H_2_O_2_ metabolism levels via MnSOD in *Arabidopsis* are unclear; thus, we used a typical oxidative stressor MV in this study, and treated mature 21-day-old seedlings of Col and *MnSOD-OE* with 5 μM MV stress for 3 days. The O_2_^•−^ and H_2_O_2_ accumulation was measured by NBT and DAB staining, respectively (Figure 5). Relative O_2_^•−^ accumulation was increased under MV treatment in Col, but decreased significantly in *MnSOD-OE* plants (Figure 5A). Relative H_2_O_2_ accumulation was increased in both Col and *MnSOD-OE* seedlings under MV treatment, but *MnSOD-OE* seedlings exhibited lower H_2_O_2_ levels (Figure 5B). These results indicated that mature *MnSOD-OE* plants retain higher superoxide scavenging ability with decreased O_2_^•−^ and H_2_O_2_ amounts.

### 2.6. Root-Length Phenotype of AtMTM1- and AtMTM2-Mutated Seedlings Analysed by Extra Mn Supply

**Figure 6:**
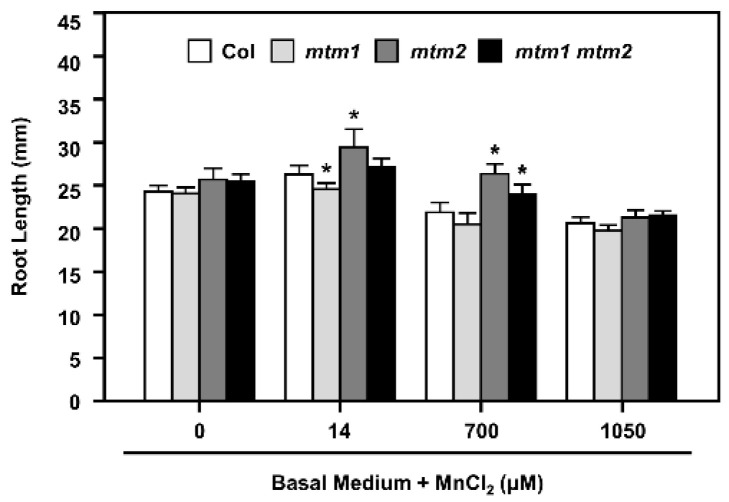
Root lengths of Col, *mtm1*, *mtm2*, and *mtm1 mtm2*-double mutants with Mn deficiency or by extra Mn supply. Seedlings were grown on the basal medium plates without MnCl_2_ supply or with extra MnCl_2_ of 14 μM (normal Mn), 700 μM (50-fold increase), or 1050 μM (75-fold increase) for 6 days, then the root lengths were measured. Data are mean ± SD of three independent repeats. *n* = 30 seedlings. * Significant at *p* < 0.05 compared with the Col.

The importance of the Mn carrier proteins, AtMTM1 and AtMTM2, for mitochondrial MnSOD activation has been reported using the miRNA-mediated *AtMTM1*-knockdown mutant (*mtm1*), the T-DNA insertional *AtMTM2*-knockout mutant (*mtm2*), and *mtm1 mtm2*-double mutants [30]. We have reported that the defective root-length phenotypes of *mtm1* and *mtm2* single and double mutant seedlings grown in 1/2 MS plates are restored through MnCl_2_ (Mn) treatment, and AtMTM1 and AtMTM2 are involved in the root-length control with divergent effects [30]. To further examine the effect of defective Mn and increased Mn supply on MnSOD activity in *mtm1*, *mtm2*, and *mtm1 mtm2*-double mutant plants, we monitored the primary root lengths on basal medium plates without Mn (Mn-deficient) or with Mn at 14 μM (normal Mn), 700 μM (50-fold increase), or 1050 μM (75-fold increase) for 6 days (Figure 6).

Col plants and all mutant lines showed similar root lengths under Mn-deficient conditions. A normal Mn supply resulted in abnormal root length in the single mutants; *mtm1* and *mtm2* plants showed shorter and longer root-length phenotypes, respectively. A 50-fold increase in Mn supply inhibited root growth, but *mtm2* and *mtm1 mtm2*-double mutant plants retained longer root lengths compared to Col plants. A 75-fold increase in Mn supply inhibited root growth in all seedlings. Based on the toxicity of increased Mn concentration and its inhibition of root growth in Col seedlings, the altered root lengths of mutant plants indicated that AtMTM1 participates more in the control of Mn homeostasis than AtMTM2. Overall, these results confirmed that AtMTM1 and AtMTM2 coordinate Mn homeostasis with divergence.

### 2.7. MnSOD Activity in AtMTM1- and AtMTM2-Mutated Protoplasts after Fe Chelation

**Figure 7:**
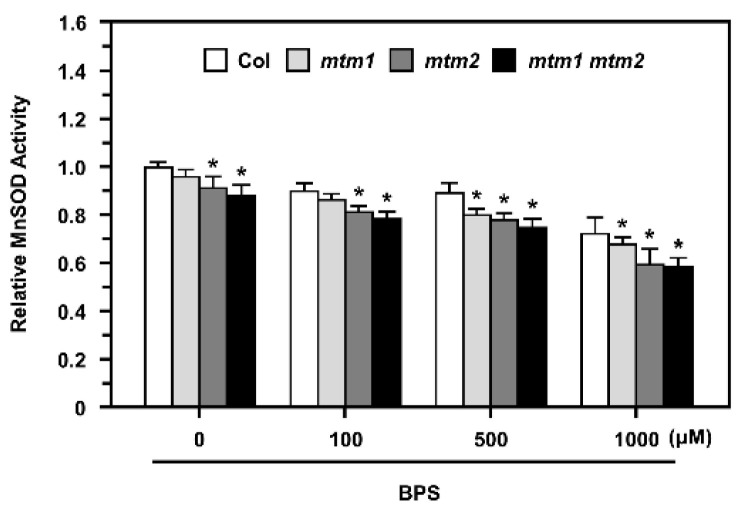
MnSOD activity of Col, *mtm1*, *mtm2*, and *mtm1 mtm2*-double mutant protoplasts after Fe^2+^-specific chelator (BPS) treatment. Protoplasts were treated without or with 100, 500, or 1000 μM BPS for 16 h. MnSOD activity in protoplasts was measured relative to Col without BPS treatment. Data are mean ± SD of three independent repeats. * Significant at *p* < 0.05 compared with the Col.

Our previous study showed that AtMTM1 and AtMTM2 are necessary for AtMnSOD activation; *AtMTM1*-mutant plants have lower Fe levels in the roots, but *AtMTM2*-mutant plants have similar Fe levels in the shoots compared to wild-type plants [30]. To delineate the role of Fe in MnSOD activation in *mtm1*, *mtm2*, and *mtm1 mtm2*-double mutants, we applied the Fe^2+^-specific chelator, BPS, to mesophyll protoplasts at 100 to 1000 μM for 16 h (Figure 7).

The *mtm1 mtm2*-double mutant protoplasts showed lower MnSOD activity than Col protoplasts without treatment, which is consistent with the results of a previous study [30]. In this study, we revealed that *mtm1* and *mtm2* protoplasts exhibited slightly decreased MnSOD activity compared to Col protoplasts. The 100 and 500 μM BPS treatments slightly inhibited MnSOD activity in Col protoplasts, and the 1000 μM BPS treatment markedly decreased MnSOD activity. By comparing relative MnSOD activity within each treatment, *mtm1* mutant protoplasts exhibited decreased MnSOD activity after 500 μM BPS treatment, and *mtm2* and *mtm1 mtm2*-double mutant protoplasts showed significantly decreased MnSOD activity from 100 μM BPS, implying that AtMTM2 was more sensitive to BPS treatment than AtMTM1. Overall, these results indicated that the altered Fe homeostasis is involved in *AtMTM1* and *AtMTM2*-mediated MnSOD activity.

### 2.8. Fe/Mn Ratio in AtMTM1- and AtMTM2-Mutant Seedlings Treated with Methyl Viologen (MV)

**Figure 8:**
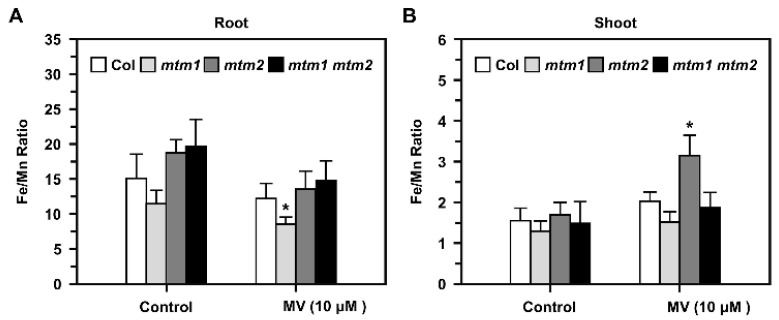
Fe/Mn ratios in roots and shoots of Col, *mtm1*, *mtm2*, and *mtm1 mtm2*-double mutants in response to MV. (**A**,**B**) Fourteen-day-old seedlings were incubated with 10 μM MV for 3 days. Fe and Mn contents in roots and shoots were measured by ICP-OES, and converted to Fe/Mn ratio. Data are mean ± SD of three independent repeats. * Significant at *p* < 0.05 compared with the Col.

We further elucidated the balance between Mn and Fe in *mtm1*, *mtm2*, and *mtm1 mtm2*-double mutants using inductively coupled plasma-optical emission spectrometry (ICP-OES). To reveal the Fe/Mn ratio in root and shoot tissues, we used MV as a representative stressor, and applied higher MV dosage with long-term treatment in this study, since *AtMTM1* and *AtMTM2* was altered slightly in short-term MV treatment as shown in Figure 2. Fourteen-day-old seedlings were incubated in 1/2 MS liquid medium containing 10 μM MV with agitation for 3 days (Figure 8). Before treatment, the Fe/Mn ratio was lower in the roots of *mtm1*, but higher in the roots of *mtm2* and *mtm1 mtm2*-double mutants compared to the ratio in Col seedlings. In addition, the Fe/Mn ratios were similar in the shoots of all seedlings. However, MV-induced stress caused a decrease in the Fe/Mn ratio in the roots of *mtm1* seedlings (Figure 8A) and an increase in the Fe/Mn ratio in the shoots of *mtm2* seedlings compared to the ratios in Col seedlings (Figure 8B). Taken together, these findings indicated that AtMTM1 and AtMTM2 are involved in Fe/Mn balance in the roots and shoots, respectively.

## 3. Discussion

The plant mitochondrial carrier family (MCF) contains approximately 60 proteins that coordinate metabolic and ionic homeostasis between the cytosol and mitochondria [36,37,38,39]. The mitochondrial MCF proteins, AtMTM1 and AtMTM2, are Mn-specific carrier proteins involved in MnSOD activation in *Arabidopsis* [30,31]. In a previous study, yeast cytosol-localised MnSOD was inactive, but its activity could be restored by treatment with a high concentration of Mn [27], and mitochondrial MnSOD was markedly enhanced by Mn treatment. Moreover, wild-type yeast cells treated with Fe show increased mitochondrial Fe levels and MnSOD protein levels, but retain normal MnSOD activity [29]. In this study, MnSOD activity was largely increased under higher concentrations of Mn treatment (Figure 1). This result indicated the Mn cofactor specificity of *Arabidopsis* MnSOD activation and the increased apoprotein levels in *MnSOD-OE* plants occurred through Mn treatment. It is worthy to adjust metal concentration based on the normal range in 1/2 MS media. In addition, we observed antagonisms between Mn and other metals, including Fe, Cu, and Zn, for MnSOD activity in *Arabidopsis*, which agrees with the results of an earlier study on the effect of Mn and Fe on MnSOD activity in *E. coli* [19].

It has been reported that *AtMTM1* is an oxidation-responsive gene [31,40]. In this study, we showed that both *AtMTM1* and *AtMTM2* gene expression levels respond to MV, NaCl, H_2_O_2_, and t-BH-induced oxidative stress in Col plants (Figure 2), and that elevated *AtMTM2* gene expression levels can be detected earlier than elevated *AtMTM1* levels. In addition, *MnSOD-OE* plants exhibited significantly enhanced MnSOD activity, but *msd1* plants showed slightly decreased MnSOD activity compared to MnSOD activity in Col plants under MV and NaCl treatments (Figure 3), indicating the post-translational regulation of MnSOD. It is worthy to investigate the post-translation regulation such as phosphorylation or ubiquitination, in order to elucidate the mechanism of MnSOD activation. In a previous study, *AtMnSOD* antisense plants displayed shorter root lengths under the high-stringency oxidative stress conditions of 0.5 μM MV [14]. The *AtMnSOD* mutant, *oiwa-1*, shows defective embryo sac formation, but the auxin gradient is not altered in this mutant [34,35]. Plants with *AtMnSOD* overexpression driven by the seed-specific promoter, At2S3, have higher germination rates after 10 μM MV and 10 mM H_2_O_2_ treatment [13]. In this study, we examined the root growth phenotype of 7-day-old *MnSOD-OE* and *msd1* seedlings on plates containing 5 nM MV or 50 mM NaCl (Figure 4). The different root lengths reflected the participation of MnSOD in early root growth. However, after transferring 5-day-old seedlings from 1/2 MS to higher-stringency conditions for 3 days, both *MnSOD-OE* and *msd1* seedlings showed longer roots. Thus, it is worth monitoring the root length for a longer period. Since MnSOD is the only reported enzyme that has the ability to catalyse superoxide (O_2_^•−^) in mitochondria, we quantified O_2_^•−^ and H_2_O_2_ levels in mature (21-day-old) *MnSOD-OE* seedlings, and demonstrated that the overexpression of MnSOD enhanced the superoxide scavenging ability and maintained the cellular levels of H_2_O_2_ (Figure 5). Overall, we demonstrated that MnSOD is crucial for the control of early root growth under stress conditions and that it scavenges superoxide radicals during plant development. Moreover, it is worthy to elucidate MnSOD-mediated oxidative stress and the accompanied shorter primary root length, as well as the phenotypes of branch roots and root hairs.

In this study, we used basal medium, with and without Mn treatment, and monitored the root length of *AtMTM1* and *AtMTM2* single and double mutants (Figure 6). The results reflected the different sensitivities of AtMTM1 and AtMTM2 to Mn, as previously described [30]. These experiments revealed that *mtm1* had a greater ability than *mtm2* to restore root length in the presence of Mn, indicating the importance of AtMTM1 in root growth. It is worthy to investigate the relationship of the primary root phenotype between *msd1* seedlings and *mtm1 mtm2*-double mutant, since both plants shared the similar longer primary root length. In a previous study, we used *mtm1 mtm2*-double mutant protoplasts to show that the mitochondrial carrier proteins, AtMTM1 and AtMTM2, are crucial for MnSOD activation [30]. In this study, we found that *AtMTM1* and *AtMTM2*-single mutant protoplasts exhibited slightly lower MnSOD activity than control protoplasts without treatment. These findings increased the evidence for the physiological roles of these carrier proteins, and indicated that AtMTM1 and AtMTM2 have different sensitivities for MnSOD activation. Moreover, we applied an Fe chelator and found that MnSOD activity was lower in *mtm2* than in *mtm1* plants, and it clearly decreased in *mtm1 mtm2*-double mutant protoplasts (Figure 7). These results indicated that AtMTM1 and AtMTM2-mediated MnSOD activation is affected by Fe homeostasis, and we suggested that the disrupted systems by Fe homeostasis include MnSOD apoprotein synthesis in the cytosol, Mn binding to MnSOD via mitochondrial AtMTM1 and AtMTM2, or tetrameric MnSOD activation in the mitochondrial matrix.

It has been reported that the yeast *MnSOD* promoter region contains heme and stress-related regulatory sites, and that *MnSOD* transcription is regulated by heme [41]. The associations between mitochondrial MnSOD levels; Fe levels; the expression levels of the Fe/S cluster biogenesis genes, *MRS3*, *MRS4*, *SSQ1*, *GRX5*, and *YFH1*, have been investigated [29,42], and elevated mitochondrial Fe levels do not correlate well with MnSOD activity in yeast. Since mitochondrial heme synthesis and Fe-S cluster biogenesis involve Fe utilisation and Fe homeostasis [33,43], we suggest that *Arabidopsis* MnSOD transportation or activation via AtMTM1 and AtMTM2 are affected by unknown Fe-S proteins. Moreover, a previous study of the Fe/Mn ratio in plants revealed an antagonistic relationship between Mn and Fe levels in root absorption and translocation from roots to shoots [16,44]. In this study, we observed that the Fe/Mn ratio was altered in the roots of *mtm1* plants and the shoots of *mtm2* plants under MV stress (Figure 8), implying physiological roles of AtMTM1 and AtMTM2 in Fe/Mn balance. It is possible that AtMTM1 or AtMTM2 are also involved in Fe regulation, and it is worth monitoring the root length of *mtm1* and *mtm2* plants grown on the basal medium with Fe added.

## 4. Materials and Methods

### 4.1. Plants and Growth Conditions

*Arabidopsis thaliana* accession Columbia-0 (Col) was the wild-type plant. *AtMnSOD* heterozygote T-DNA-inserted knockdown mutant (*msd1*; SALK 122275) was requested from Arabidopsis Biological Resource Center (ABRC). *mtm1* (miRNA-mediated *AtMTM1*-knockdown), *mtm2* (T-DNA insertional *AtMTM2*-knockout), and *mtm1 mtm2*-double mutants were established in our previous study [30]. Plants were grown in soil or on 1/2 MS (Sigma-Aldrich, St. Louis, MO, USA) plates supplied with 1% sucrose and 0.8% Phytagel (Sigma-Aldrich) with illumination at 80–100 μmol m^−2^ s^−1^ under standard long day condition (16 h light/8 h dark) at 22–24 °C.

### 4.2. Generation of AtMnSOD-Overexpressing Plants

The coding region of *Arabidopsis AtMnSOD* was amplified by RT-PCR and ligated into the yT&A vector (Yeastern Biotech, Taipei, Taiwan) for sequencing, then subcloned into the *Sac*I and *Xba*I sites of the destination vector pPZP200GB with CaMV *35S* promoter [45]. *AtMnSOD*-overexpressing (*MnSOD-OE*) plants were generated by *Agrobacterium tumefaciens* GV3101-mediated transformation and the floral dip method [46], then selected by Basta.

### 4.3. Genotyping, RT-qPCR, In-Gel SOD Activity, and Immunoblotting Assay

Plant genomic DNA was extracted for genotyping by PCR, as previously described [47]. Total RNA was prepared with TRIZOL reagent (Invitrogen) and TURBO DNA-free Kit (Applied Biosystems, Foster City, CA, USA). cDNA synthesis was performed by High-Capacity cDNA Reverse Transcription Kits (Applied Biosystems). Transcription levels were monitored by RT-qPCR with KAPA SYBR FAST q-PCR Kit (KAPA Biosystems, Wilmington, MA, USA). *Protein Phosphatase 2A subunit A3* (*PP2A*) was an internal control [48,49] for RT-qPCR.

Fourteen-day-old seedlings were incubated in 1/2 MS liquid medium containing MnCl_2_ (Mn), Fe citrate (Fe), Mn plus Fe, or oxidative stressors of methyl viologen (MV), NaCl, H_2_O_2_, or tert-butyl hydroperoxide (t-BH) with agitation. The gene expression levels were analyzed by q-PCR. *AOX1A*, a mitochondrial stress gene, was a reference [30].

Protein was extracted by ice-cold griding buffer (150 mM Tris-HCl, pH 7.2), and supernatant was purified twice by centrifugation for 10 min at 16,000× *g* at 4 °C [30]. Protein concentration was determined by Bio-Rad protein assay reagent (Bio-Rad, Techview, Singapore). In-gel SOD activity assay was performed as previously described [50]. MnSOD protein level was examined by immunoblotting with α-MnSOD (Agrisera, Västerbäck, Vännäs, Sweden) antibody. Actin protein was used as an internal control and analyzed with α-Actin (Agrisera) antibody.

### 4.4. Analysis of O_2_^•−^ and H_2_O_2_ Accumulations by Nitrobule Tetrazolium (NBT) and Diaminobenzidine (DAB) Staining

Twenty-one-day-old seedlings were incubated in 1/2 MS liquid medium containing 5 μM MV with gentle shaking for 3 days, then stained with 1 mg/mL NBT or 1 mg/mL DAB in dark overnight. Leaves were fixed with decolorizing solution containing ethanol, lactic acid, and glycerol (3:1:1) for 1 h, then washed with 70% ethanol for 1 h to remove chlorophyll completely. O_2_^•−^ and H_2_O_2_ accumulation were detected by NBT and DAB staining, respectively [51,52,53]. The relative accumulation was quantified by image J system (https://imagej.nih.gov/ij/download.html accessed on 24 February 2022).

### 4.5. Root Length Assay in Response to Oxidative Stressors and Mn Treatment

The root lengths of 7-day-old seedlings on 1/2 MS plates containing mild-stringency stressors of 5 nM MV or 50 mM NaCl were measured. In addition, 5-day-old seedlings were transferred from 1/2 MS medium to plates containing higher-stringency stressors of 10 nM MV or 150 mM NaCl for 3 days were conducted.

Moreover, the root lengths of seedlings in the Mn deficiency condition were analyzed by using the basal medium containing 5 mM KNO_3_, 2 mM MgSO_4_, 2 mM Ca (NO_3_)_2_, 2.5 mM KH_2_PO_4_, 70 μM H_3_BO_3_, 40 μM Fe EDTA, 1 μM ZnSO_4_, 0.5 μM CuSO_4_, 0.2 μM Na_2_MoO_4_, 4.7 mM MES (pH 5.5) with 43 mM sucrose, then solidified with 0.8% Phytagel [54]. Seedlings were grown on plates supplied without MnCl_2_ (Mn deficient) or with MnCl_2_ at 14 μM (normal Mn), 700 μM (50-fold increase), or 1050 μM (75-fold increase) for 6 days.

### 4.6. Fe chelation in Arabidopsis Protoplasts

The Fe^2+^-specific chelator bathophenanthroline disulfonate, BPS (Sigma-Aldrich), can decrease Fe contents in yeast cytosol and mitochondria, and the effect of BPS on MnSOD activity was analyzed [29,42,55]. *Arabidopsis* mesophyll protoplasts were isolated from four-week-old plants [56]. An amount of 3 × 10^5^ protoplasts were treated with BPS at 100 μM, 500 μM, or 1000 μM for 16 h [55,57,58], and were examined for its effect on MnSOD activity.

### 4.7. Fe/Mn Ratios in Roots and Shoots in Response to MV

The inductively coupled plasma-optical emission spectrometry (ICP-OES) (PerkinElmer OPTIMA 5300) analysis was applied to measure Mn and Fe metal contents in plants [59,60]. Fourteen-day-old seedlings were incubated in 1/2 MS liquid medium containing 10 μM MV with agitation for 3 days, then shoots and roots were separated. An amount of 0.1 g dried samples were used for ICP-OES analysis [30]. Spinach and tomato leaves (NIST SRM-1570a and NIST SRM-1573a) were the references. The output Mn and Fe contents by ICP-OES were converted to Fe/Mn ratio.

### 4.8. Statistical Analysis

All experiments were repeated independently at least three times. Statistical analysis involved Student’s *t*-test and Duncan’s multiple range test. *p* value < 0.05 was considered statistically significant.

### 4.9. PCR Primers and GenBank Accession Numbers

Primers and gene accession numbers are listed in Supplementary Table S1.

## 5. Conclusions

This study strengthened the importance of MnSOD activation through its carrier proteins AtMTM1 and AtMYM2 by metal ion treatments and oxidative stressors. We showed that MnSOD activity was specifically enhanced by Mn treatment, and antagonism occurred between Mn and other metals for MnSOD activation. We clarified the post-translational regulation of MnSOD during oxidative stress and demonstrated that MnSOD participates in the control of early root growth and enhances superoxide scavenging efficiency in mature seedlings. It is worthy to connect the altered root-length phenotype and elevated MnSOD enzyme activity. We also revealed that altered Fe homeostasis inhibited MnSOD activity through the carrier proteins AtMTM1 and AtMTM2. Especially, AtMTM1 and AtMTM2 participate in Fe/Mn regulation with tissue specificity. It would be interesting to investigate the mechanism of MnSOD post-translational regulation through phosphorylation site mutation, and to elucidate the substrate affinity of AtMTM1 and AtMTM2 through in vitro studies.

## Data Availability

The data presented in this study are available in article and supplementary materials.

## Supplementary Materials

The following supporting information can be downloaded at: https://www.mdpi.com/article/10.3390/plants11050619/s1. Figure S1: Characterizations of *AtMnSOD*-overexpressing (*MnSOD-OE*) plant; Figure S2: Characterizations of *AtMnSOD* T-DNA knockdown (*msd1*) mutant; Figure S3: Control experiment of *AtMTM1* and *AtMTM2* gene expression levels in Col plants; Table S1: Primers for cloning, genotyping, RT-qPCR, and accession numbers of genes.
