# Supplementary material for: Impacts of Mn, Fe, and Oxidative Stressors on MnSOD Activation by AtMTM1 and AtMTM2 in Arabidopsis
Source: Plants (Basel). 2022 Feb 24;11(5):619. doi: 10.3390/plants11050619 (PMC8912514; doi:10.3390/plants11050619)
Supplement: Supplementary file 1 [file plants-11-00619-s001.zip › plants-1565283-supplementary.pdf]

Supplementary Table S1. Primers for cloning, genotyping, RT-qPCR, and accession numbers of genes.

**Purpose**	**Sequence (5'→ 3')**	**Primer Set**
Construction of *AtMnSOD-OE*	GAGCTCTTTGGCGACCACTAGAAGGAGA	*SacI*-AtMnSOD-OE-Fw
	TCTAGAAGCTGAGCTGGAACTGGTTCATCTC	*XbaI*-AtMnSOD-OE-Rv
Genotyping of *msd1*	CGATTCGTTGTGTAGCGAG	AtMnSOD-Fw (LP)
	TTCAGTTGTTTTCCTTCTCATAAAC	AtMnSOD-Rv (RP)
T-DNA left border	ATTCAGTACATTAAAAACGTCCGCAAT	BP
RT-qPCR for *AtMTM1*	AGAGGACTGTTCATGGGAATGG	AtMTM1-RT-qPCR-Fw
	ATACTTGACCACTTCGTAGAAGGAAAC	AtMTM1-RT-qPCR-Rv
RT-qPCR for *AtMTM2*	GAGCCATGACAATGACCACAAG	AtMTM2-RT-qPCR-Fw
	CTCTAGCCCCTGCTCCACTAAAC	AtMTM2-RT-qPCR-Rv
RT-qPCR for *AtMnSOD*	TGCCATTGACGCTCACTTTG	AtMnSOD-RT-qPCR-Fw
	TTGTCTAGTCCGAGCCACAC	AtMnSOD-RT-qPCR-Rv
RT-qPCR for *AtAOX1A*	GAATGTTCCTGCTCCGGCTAT	AtAOX1A-RT-qPCR-Fw
	TCAGCACGAACAACCATCACA	AtAOX1A-RT-qPCR-Rv
Internal control of RT-qPCR	CCTGCGGTAATAACTGCATCT	AtPP2A-RT-qPCR-Fw
	CTTCACTTAGCTCCACCAAGCA	AtPP2A-RT-qPCR-Rv

Sequence data in this article can be found in the TAIR database under the following accession numbers: *AtAOX1A* (At3G22370), *AtMnSOD* (*AtMSD1*; At3G10920), *AtMTM1* (At4G27940), *AtMTM2* (At2G46320), *AtPP2A* (At1G13320), and *AtUBQ10* (At4G05320).

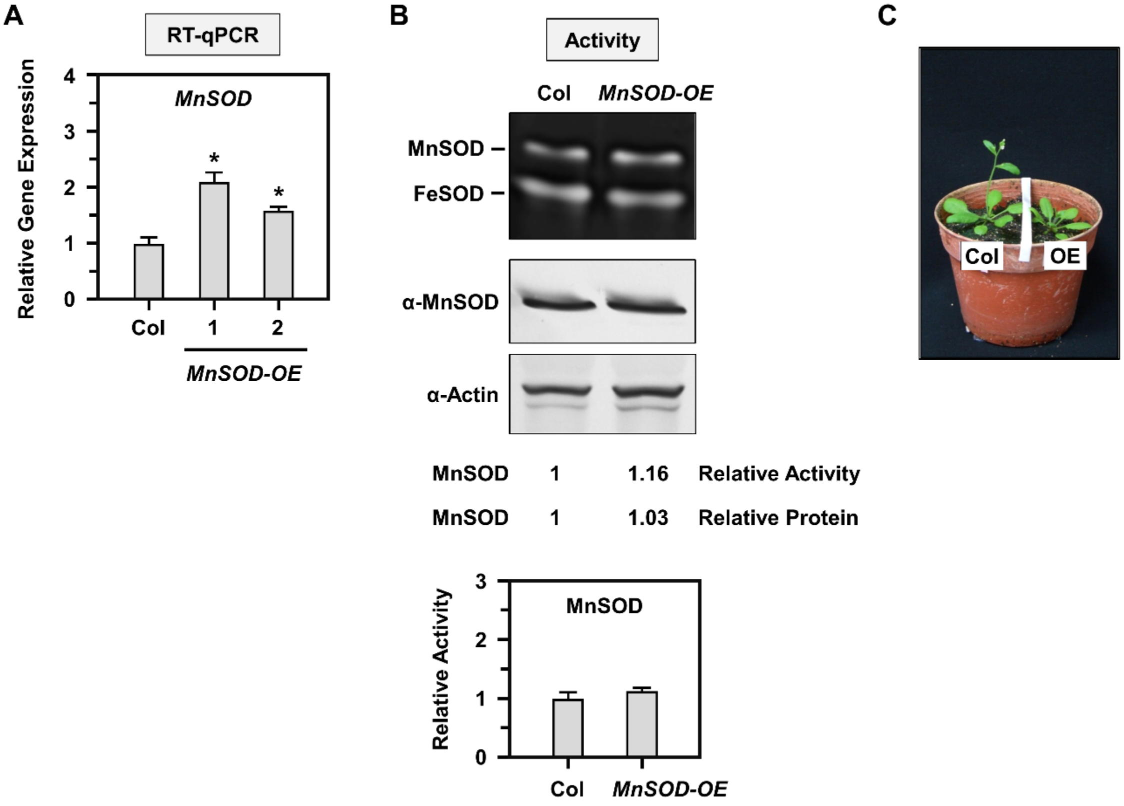

Supplementary Figure S1. Characterizations of *AtMnSOD*-overexpressing (*MnSOD-OE*) plant. **(A)** *AtMnSOD* gene expression in Col and *MnSOD-OE* seedlings was analyzed by RT-qPCR. *PP2A* was used as internal control. *AtMnSOD* gene expression level in *MnSOD-OE* was measured relative to that in Col. Data are mean ± SD of three biological replicates. * Significant at $P < 0.05$. **(B)** In-gel SOD activity assay (top) and immunoblotting with α-MnSOD and α-Actin antibodies (bottom). Actin was used as a loading control. MnSOD activity and protein levels in *MnSOD-OE* were measured relative to that in Col. **(D)** Late-flowering phenotype of one-month-old *MnSOD-OE* plant.

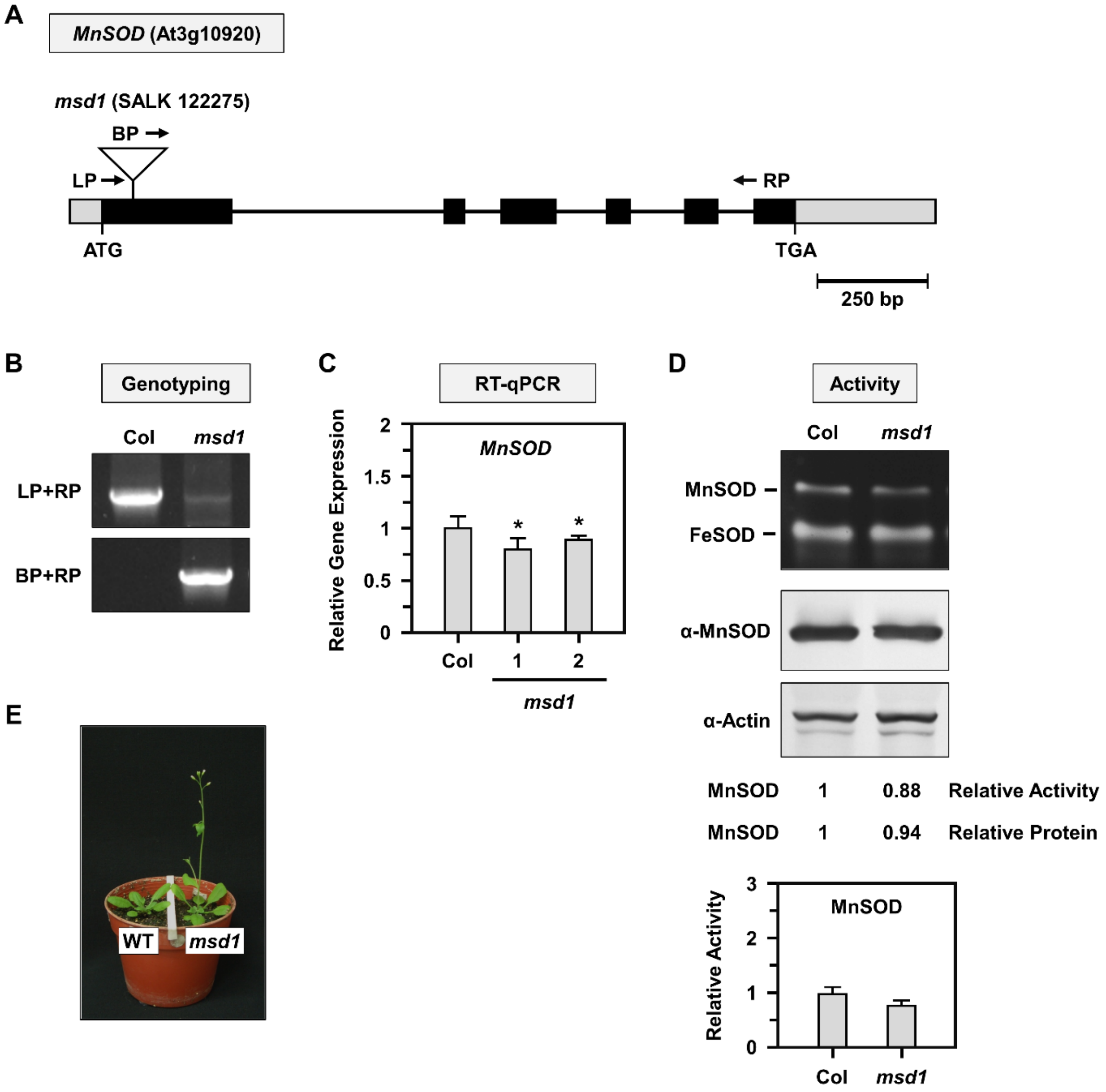

Supplementary Figure S2. Characterizations of *AtMnSOD* T-DNA knockdown (*msd1*) mutant. **(A)** Schematic map of *AtMnSOD* gene (At3G10920), T-DNA insertion site, and primers (arrows) used for genotyping. **(B)** Genotyping by PCR in *msd1* was performed with DNA flanking sequence primers (LP and RP) and T-DNA border primer (BP). **(C)** The *AtMnSOD* gene expression level in Col and *msd1* mutant was analyzed by RT-qPCR. *PP2A* was used as internal control. *AtMnSOD* gene expression level in *msd1* was measured relative to that in Col. Data are mean ± SD of three biological replicates. * Significant at $P < 0.05$. **(D)** In-gel SOD activity assay (top) and immunoblotting with α-MnSOD and α-Actin antibodies (middle and bottom). Actin was used as a loading control. MnSOD activity and protein levels in *msd1* were measured relative to that in Col. **(F)** Early-flowering phenotype of one-month-old *msd1* mutant.

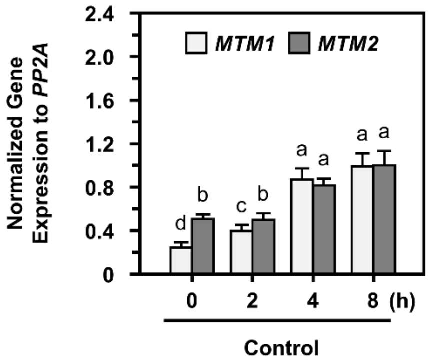

Supplementary Figure S3. Control experiment of *AtMTM1* and *AtMTM2* gene expression levels in Col. 14-day-old seedlings were incubated in 1/2 MS liquid medium without stressor treatment. *PP2A* was used as internal control. Data are mean ± SD of three biological replicates. The statistical significances ($P < 0.05$) are indicated as different letters (Duncan's multiple range test).
